# Effect of vitamin D supplementation during pregnancy on mid-to-late gestational blood pressure in a randomized controlled trial in Bangladesh

**DOI:** 10.1097/HJH.0000000000002609

**Published:** 2020-08-10

**Authors:** Anita Subramanian, Jill Korsiak, Kellie E. Murphy, Abdullah Al Mahmud, Daniel E. Roth, Alison D. Gernand

**Affiliations:** a Department of Nutritional Sciences, The Pennsylvania State University, University Park, Pennsylvania, USA; b Centre for Global Child Health, Hospital for Sick Children; c Department of Pediatrics, University of Toronto; d Department of Obstetrics and Gynecology, Mt. Sinai Hospital, Toronto, Canada; e Nutrition and Clinical Services Division, icddr,b, Dhaka, Bangladesh

**Keywords:** 25-hydroxyvitamin D, arterial pressure, cholecalciferol, hemodynamics, pregnancy

## Abstract

To examine the dose-dependent effect of maternal vitamin D during pregnancy on blood pressure from mid-to-late gestation within the context of a randomized, placebo-controlled trial of vitamin D supplementation in Bangladesh (*n* = 1298).

**Methods:**

Healthy women without hypertension were enrolled at 17–24 weeks gestation and randomized to one of four vitamin D doses during pregnancy: placebo, 4200, 16 800 or 28 000 IU/week. This substudy examined 1257 women with blood pressure measured at enrollment with at least one other timepoint (measurements included at 24 weeks, 30 weeks, and weekly from 36 weeks until delivery). Effects of vitamin D on SBP or DBP were analyzed using mixed-effects models.

**Results:**

Vitamin D did not have an effect on SBP or DBP at 24 or 30 weeks; blood pressure was higher at 36 weeks for the highest dose versus placebo [mean difference (95% CI) mmHg: SBP = 2.3 (0.9–3.7); DBP = 1.9 (0.7–3.0)]. The differences in changes in SBP and DBP between vitamin D groups and placebo across intervals were small (*P* > 0.10), but the difference for 28 000 IU/week versus placebo was the highest from 30 to 36 weeks [SBP 0.2 (−0.1 to 0.5) and DBP 0.2 (−0.0 to 0.4) mmHg].

**Conclusion:**

Vitamin D supplementation starting mid-pregnancy did not affect SBP or DBP until late gestation, and then only at the highest dose. These results do not support the clinical use of vitamin D in pregnancy to lower maternal blood pressure.

## INTRODUCTION

Preeclampsia is estimated to complicate 2–8% of pregnancies worldwide but women living in low-income countries are at a greater risk of adverse pregnancy outcomes because of limited access to high-quality prenatal care [1]. About 9% of maternal deaths in Asia and Africa are attributed to hypertensive disorders compared with 25% in Latin America and the Caribbean [2]. Preeclampsia is associated with preterm and small-for-gestational age (SGA) births [1,3]. Gestational hypertension usually results in milder adverse outcomes than preeclampsia [4]; however, women with gestational hypertension are at a greater risk for developing preeclampsia [5]. Women with gestational hypertension or preeclampsia have an increased risk of developing hypertension and cardiovascular diseases later in life [6].

Although best known for bone health [7], vitamin D has also been linked to many poor health outcomes including risk of hypertensive disorders of pregnancy [8]. The risk of preeclampsia was found to be three to five times higher among women with vitamin D deficiency in Dhaka, Bangladesh [9]. Among Swedish women, odds of preeclampsia were lower when circulating concentrations of 25-hydroxyvitamin D [25(OH)D] increased to at least 30 nmol/l from the first to third trimester [10]. Mixed findings have been reported for the association between vitamin D and gestational hypertension [8].

Blood pressure normally declines from conception to 18–20 weeks gestation followed by an increase until delivery [11,12]. The increase in blood pressure after mid-gestation is estimated to be about 7% in SBP and 9% in DBP among normotensive women [12]. Blood pressure rises rapidly during the third trimester, and the mean increase among normotensive women from 36 weeks until delivery has been reported to be 1.1 mmHg in SBP and 1.3 mmHg in DBP [11]. Factors related to the pattern of blood pressure change across pregnancy include maternal age [11], prepregnancy BMI [11], parity [11], and gestational weight gain [13]. Overall, the patterns of blood pressure changes in pregnancy have been examined in normal and complicated pregnancies in high-income countries, but not among women in low-resource settings.

Few vitamin D studies have reported gestational hypertension as an outcome, and most reporting preeclampsia have included it as a secondary outcome [8]. Observational studies have reported inconsistent findings on the associations between vitamin D and hypertensive disorders of pregnancy, and notably there are substantial limitations to most of these studies [8]. Higher risk of gestational hypertension was associated with increasing concentrations of 25(OH)D among women enrolled in Project Viva; for every increase in 25(OH)D by 25 nmol/l, the odds increased by 1.32 (95% CI 1.01 to 1.72) [14]. In contrast, a recent meta-analysis of randomized controlled trials of vitamin D supplementation during pregnancy did not report an effect of vitamin D on preeclampsia or gestational hypertension [15]. The latest Cochrane review found that vitamin D lowered the risk of preeclampsia but did not impact the risk of gestational hypertension [16]. Largely missing from these studies is an investigation of blood pressure itself. Only a few small studies have examined blood pressure changes in response to vitamin D supplementation [17–19]. The objective of this study was to examine the dose-dependent effect of maternal vitamin D supplementation during pregnancy on blood pressure from mid-to-late gestation in the context of a large randomized controlled trial in Bangladesh. This was a post hoc analysis that was not part of the original study design.

## METHODS

The current study examining blood pressure was conducted with data from the Maternal Vitamin D for Infant Growth (MDIG) trial, a randomized, placebo-controlled, dose-ranging trial of vitamin D supplementation in Dhaka, Bangladesh [20]. The primary objective of the parent trial was to examine if maternal vitamin D supplementation versus placebo impacts infant length [21]. In brief, the MDIG trial included 1298 pregnant women between 17 and 24 completed weeks of gestation at the Maternal and Child Health Training Institute in Dhaka. Participants were individually randomized to one of four vitamin D doses during pregnancy: placebo (0 IU/week), low (4200 IU/week), middle (16800 IU/week) or high (28000 IU/week). In addition, all participants also received calcium (500 mg/day) and iron and folic acid (66 mg iron/day; 350 μg folic acid/day) supplements throughout the intervention period. Vitamin D supplements were administered weekly by study personnel, who also directly observed tablet ingestion during home visits. Adherence to study supplements across all groups was at least 90% during pregnancy [20]. The trial findings showed a clear dose–response effect of vitamin D supplementation on the concentrations of maternal 25(OH)D [20]. All participants provided written informed consent. The MDIG trial received ethical approval from The Hospital for Sick Children Research Ethics Board, and the International Centre for Diarrhoeal Disease Research, Bangladesh (icddr,b).

The MDIG trial enrolled pregnant women who were generally in good health; 18 years of age and above; had completed 17–24 weeks of gestation based on recalled last menstrual period (LMP) date and ultrasound scan; and had a singleton gestation. Women were excluded if they presented a history of any medical condition; preexisting hypertension defined as SBP at least 140 mmHg and/or DBP at least 90 mmHg; moderate-to-severe proteinuria (>300 mg/dl or 3+/4+ on urine dipstick); severe anemia (hemoglobin <70 g/l); or high-risk pregnancy, such as multiple gestation or major congenital anomaly. Additional details of methods and eligibility are published [21]. Of note, we are not aware of any pregnancies because of assisted reproductive technologies in this low-resource setting.

In the current study, we selected all participants enrolled in the parent trial who had a blood pressure measurement at baseline (*n* = 1292) and at least one subsequent measurement before the onset of labor, yielding an analytic sample size of 1257 women. The number of women across treatment groups were: placebo, *n* = 250; 4200 IU/week, *n* = 252; 16800 IU/week, *n* = 251; and 28 000 IU/week, *n* = 504. The highest dose group included participants randomized to continue to receive 28 000 IU/week for 6 months postpartum and those who received placebo postpartum, and therefore, included approximately double the number of participants as each of the other three groups.

### Blood pressure measurements

Maternal blood pressure was measured using an automated digital blood pressure monitor (Microlife BP 3MX1–1) by research staff at the hospital or at home visits. The Microlife WatchBP Home (BP 3MX1–1) has been previously validated for blood pressure measurement in pregnancy [22]. Women rested for 5 min and then an appropriately sized cuff was placed on the left arm. The cuff size (small, medium, large) was determined by measuring the participants arm circumference at approximately 2–3 cm above the elbow on the left arm. Women were seated in an upright position with their feet flat on the floor and left arm on a table such that the cuff was at the same height as the heart. Two measurements for SBP and DBP were taken at least 1 min apart. If the difference between two SBP or DBP measurements differed by greater than 10 mmHg, a third reading was taken at least 1 min after the second measurement.

Blood pressure was measured at screening and enrollment, followed by measurements at 24 and 30 weeks, and then weekly from 36 weeks until delivery. Study visits were not always timed exactly to these gestational ages. Measurements obtained during labor were excluded from the present analysis as blood pressure during the intrapartum period can be affected by stressors, which might not be reflective of blood pressure during gestation.

### Blood pressure outcomes

SBP or DBP at each visit was the mean of two readings. Whenever three measurements were available, we used the mean of the two measurements that were closest to one another. If only one measurement was available for SBP (38 occurrences) and/or DBP (40 occurrences), then it was included in the analysis. When pulse pressure (difference between SBP and DBP) was less than 10 mmHg (20 occurrences), it was considered biologically implausible and excluded from the analysis. Low blood pressure was defined as DBP less than 60 mmHg [23].

### Maternal characteristics

Information was collected by structured interviews including age, marital status, education, occupation, and parity. Household-level questions assessed electricity and ownership of a television, refrigerator or mobile phone. Gestational age was estimated using recalled LMP date and ultrasound at enrollment. We measured weight, height, and blood concentrations of hemoglobin, 25(OH)D and calcium. Further details are reported elsewhere [21].

### Statistical analysis

Maternal characteristics were presented as means/medians for continuous variables and as percentages for categorical variables. Differences in baseline characteristics across treatment groups at enrollment were examined using ANOVA for continuous variables, and chi-squared tests or Fischer's exact test for categorical variables. Normality of distributions was checked using kernel density and quantile–quantile (Q-Q) plots. Bivariate relationships were visualized using scatter plots with locally weighted regression (LOWESS).

We fit linear mixed-effects models to estimate the effect of vitamin D treatment on SBP or DBP (separate outcomes) longitudinally from mid-to-late pregnancy. These models account for blood pressure measurements after enrollment with the first set of measurements at ∼24 weeks of gestation, 4 weeks on an average after starting supplementation. The mixed-effects model also accounts for unequally spaced repeated measurements within women at varying time points across gestation and has the flexibility to include women with missing observations. To account for the nonlinear changes in blood pressure across pregnancy, we used linear splines with knots at 30 and 36 weeks gestation, coinciding with scheduled timing of measurements. Models also included vitamin D treatment groups as fixed effects, participants as random intercepts, and interaction terms between treatment groups and gestational age spline terms to estimate the differences between treatment groups. Models were adjusted for baseline SBP or DBP and gestational age at enrollment. We used contrasts of margins to estimate the marginal effects of vitamin D at 24, 30 and 36 weeks gestation. We also fit the models with random slopes and findings were similar. To examine the potential role of serum calcium as a mediator of the effect of vitamin D on blood pressure, we adjusted for maternal serum calcium concentration at 30 weeks gestation.

We stratified by low blood pressure at enrollment and baseline vitamin D status, using the same models, to examine potential sub-group effects. Finally, we re-examined the effect of vitamin D on gestational hypertension, similar to the analysis previously reported in the published main MDIG trial paper (Table S27 in the supplementary appendix of ref [20]), but with a pairwise comparison between each vitamin D dose and placebo using log-binomial models to estimate relative risk.

We performed a sensitivity analysis to examine the robustness of findings by only including SBP or DBP where the difference in measurements between any two separate sets was less than 10 mmHg. We also examined vitamin D effects in term births alone. All analyses were conducted using Stata 15.0 software (StataCorp, College Station, Texas, USA).

## RESULTS

**FIGURE 1:**
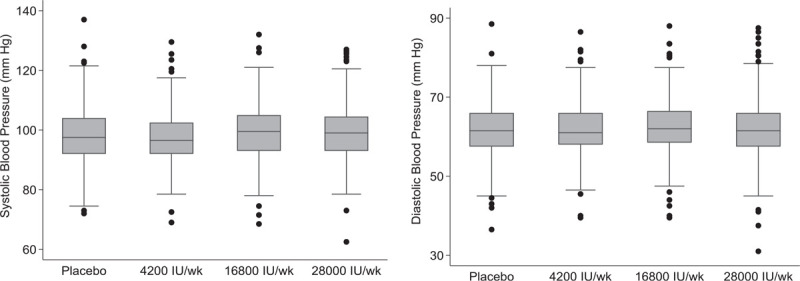
SBP and DBP across treatment groups at enrollment (17–24 weeks gestation) Each box shows the interquartile range (25th to 75th percentile); the median depicted by the line in the middle of the box; upper and lower lines or whiskers show the adjacent values (1.5× interquartile range); outliers are shown as black circles. The mean (SD) SBP (mmHg) at enrollment by treatment group was: placebo, 98.3 (10.0); 4200 IU/week, 98.0 (8.8); 16 800 IU/week, 99.1 (9.6); 28 000 IU/week, 99.2 (9.3) and DBP was placebo, 62.0 (7.1); 4200 IU/week, 62.0 (7.1); 16 800 IU/week, 62.5 (7.4); and 28 000 IU/week, 62.0 (7.2). SD, standard deviation.

Vitamin D status, maternal and household characteristics were all similar across treatment groups at enrollment (Table 1). There were no differences in SBP or DBP across treatment groups at enrollment (Fig. 1). The prevalence of DBP less than 60 mmHg at enrollment was 27%.

**TABLE 1:** Characteristics of pregnant women at enrollment (17–24 weeks), by treatment group^a^

	Vitamin D treatment group^b^				
Outcome characteristics	Placebo (*n* = 250)	4200 IU (*n* = 252)	16800 IU (*n* = 251)	28000 IU (*n* = 504)	*P*-value^c^
Self-reported					
Age (years)	23.5 ± 4.4	23.1 ± 4.3	23.0 ± 3.8	23.3 ± 4.2	0.425
Gestational age (weeks), median (min, max)^d^	20.4 (17, 24)	20.1 (17, 24)	20.3 (17, 24)	20.3 (17, 24)	0.847
Marital status (married)^e^	248 (99.2)	252 (100)	250 (100)	503 (100)	0.079
Level of education completed^e^					0.645
Primary/middle school	142 (63.7)	134 (59.0)	141 (63.2)	284 (63.8)	
Secondary or higher	81 (36.3)	93 (41.0)	82 (36.8)	161 (36.2)	
Primary occupation^e^					0.927
Homemaker	233 (93.2)	235 (93.3)	235 (94.0)	474 (94.2)	
Other	17 (6.8)	17 (6.8)	15 (6.0)	29 (5.8)	
Household ownership^e^					
Has electricity	247 (98.8)	251 (99.6)	249 (99.6)	503 (100)	0.051
Owns a television	203 (81.2)	210 (83.3)	215 (86.0)	417 (82.9)	0.541
Owns a refrigerator	130 (52.0)	128 (50.8)	133 (53.2)	268 (53.3)	0.921
Owns a mobile telephone	241 (96.4)	245 (97.2)	242 (96.8)	490 (97.4)	0.847
Gravidity, median (min, max)^f^	2 (1, 9)	2 (1, 6)	2 (1, 6)	2 (1, 7)	0.809
Parity, median (min, max)^g^	1 (0, 5)	1 (0, 4)	1 (0, 4)	1 (0, 4)	0.539
Measured					
Height (cm)	151.2 ± 5.4	150.8 ± 5.0	150.8 ± 5.4	151.0 ± 5.5	0.822
Weight (kg)	54.5 ± 10.3	53.2 ± 10.2	54.0 ± 10.0	54.3 ± 9.9	0.429
Hemoglobin (g/dl)	10.6 ± 1.1	10.6 ± 1.2	10.6 ± 1.1	10.6 ± 1.1	0.857
Anemia (hemoglobin <10.5 g/dl)	103 (41.2)	111 (44.1)	108 (43.0)	206 (40.9)	0.832
SBP (mmHg)	98.3 ± 10.0	97.5 ± 8.8	99.1 ± 9.6	99.2 ± 9.3	0.091
DBP (mmHg)	61.5 ±7.1	61.8 ± 7.1	62.5 ± 7.4	61.9 ± 7.2	0.528
Pulse pressure (mmHg)^h^	36.8 ± 7.3	35.8 ± 6.5	36.7 ± 7.1	37.3 ± 6.9	0.031
Serum calcium (mmol/l)	2.26 ± 0.09	2.25 ± 0.08	2.26 ± 0.09	2.25 ± 0.09	0.079
Serum 25(OH)D (nmol/l)^e^	27.5 ± 13.7	27.4 ± 14.3	28.8 ± 14.1	26.7 ± 14.0	0.296
Vitamin D deficiency^e^^,^^i^	152 (61.3)	158 (63.0)	152 (61.0)	338 (67.3)	0.218

25(OH)D, 25-hydroxyvitamin D; IU, international unit.

a Data are mean ± SD or *n* (%) unless otherwise specified. The data in this table are similar to what has previously been reported [20].

b Vitamin D doses were administered weekly within a randomized controlled trial.

c *P* value from ANOVA for continuous variables; chi-squared test for categories; Fischer's exact test when cell count was less than 5; nonparametric equality of medians test for gestational age at enrollment, gravidity and parity.

d On the basis of date of last menstrual period and/or ultrasound.

e Two missing values for marital status; 139 missing values for education; two missing values for occupation; two missing values for household ownership; six missing values for serum 25(OH)D; six missing values for vitamin D deficiency.

f Gravidity was defined as the total number of pregnancies including the current pregnancy.

g Parity was defined as the number of previous live births.

h Pulse pressure was defined as the difference between SBP and DBP.

i Vitamin D deficiency was defined as a concentration of 25(OH)D less than 30 nmol/l.

There were no differences in mean SBP or DBP at 24 and 30 weeks for vitamin D groups compared with placebo (Table 2). Mean SBP and DBP at 36 weeks were each ∼2 mmHg higher in women receiving the highest dose of vitamin D compared with placebo (adjusting for baseline blood pressure and gestational age at enrollment), although there was no evidence of a dose-dependent effect in the low and middle doses (Table 2). When disaggregated by week of gestation, the effect of the highest dose vitamin D was apparent from weeks 35 to 38 (see Table S1, Supplemental Digital Content 1, http://links.lww.com/HJH/B433). Upon adjustment for serum calcium at 30 weeks gestation, mean SBP and DBP at 36 weeks remained higher in women receiving the highest dose of vitamin D compared with placebo but the effects were slightly attenuated [mean difference (95% CI): SBP (mmHg) = 2.20 (0.78 to 3.62); DBP (mmHg) = 1.76 (0.60 to 2.91)]. Other results adjusting for calcium were similar to main findings.

**TABLE 2:** Effect of vitamin D supplementation^a^ on maternal blood pressure at 24, 30 and 36 weeks gestation, *n* = 1257

	Mean (95% CI)	Mean difference (95% CI)^b^^,^^c^			
	Placebo	4200 IU/week^c^	16 800 IU/week^c^	28 000 IU/week^c^	*P* value^d^
SBP (mmHg)					
24 weeks	101.0 (99.7 to 102.3)	0.61 (−1.17 to 2.40)	0.50 (−1.29 to 2.29)	0.48 (−1.06 to 2.03)	0.908
30 weeks	97.7 (96.4 to 99.0)	0.36 (−1.47 to 2.19)	0.61 (−1.24 to 2.45)	1.26 (−0.33 to 2.85)	0.414
36 weeks	104.4 (103.3 to 105.6)	1.62 (0.01 to 3.22)	0.55 (−1.06 to 2.17)	2.32 (0.91 to 3.72)^e^	0.005
DBP (mmHg)					
24 weeks	65.0 (64.0 to 66.0)	0.32 (−1.12 to 1.77)	−0.28 (−1.73 to 1.18)	0.12 (−1.13 to 1.37)	0.872
30 weeks	62.5 (61.4 to 64.0)	0.09 (−1.39 to 1.58)	−0.07 (−1.57 to 1.42)	0.74 (−0.55 to 2.03)	0.510
36 weeks	68.7 (68.0 to 70.0)	0.74 (−0.56 to 2.05)	0.17 (−1.14 to 1.48)	1.86 (0.71 to 3.00)^e^	0.003

CI, confidence interval; IU, international unit.

a Vitamin D doses were administered weekly within a randomized controlled trial.

b Mixed-effects models with linear spline knots at 30 and 36 weeks gestation were used to estimate effect of vitamin D on changes in SBP or DBP. Models included spline terms for gestational age; treatment group; and interaction terms between treatment group and gestational age spline terms. Models were fit to test the effect of each vitamin D treatment group on SBP or DBP with reference to placebo. Models were adjusted for baseline SBP or DBP and gestational age at enrollment. Marginal effects of treatment group were examined on SBP or DBP at 24, 30, and 36 weeks gestation.

c Values represent mean difference in SBP or DBP for each vitamin D group compared to placebo.

d *P* value represents overall difference in means across treatment groups.

e Mean blood pressure was different between placebo and 28 000 IU/week; estimated using the contrast of margins (*P* < 0.01).

**FIGURE 2:**
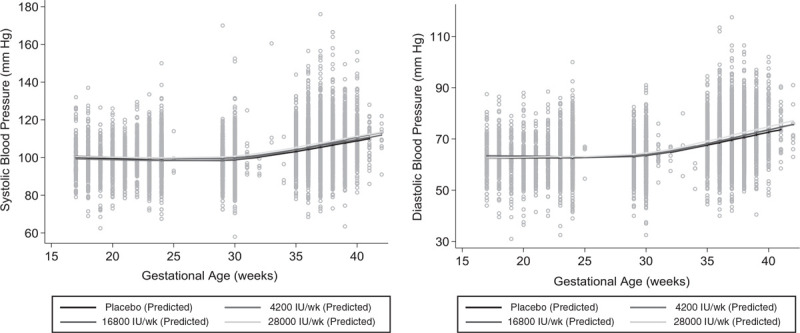
SBP and DBP across pregnancy by treatment group (placebo *n* = 250; 4200 IU *n* = 252; 16 800 IU *n* = 251; 28 000 IU *n* = 504). Lines represent predicted means estimated by mixed-effects models with cubic spline knots at 24, 30, and 36 weeks gestation (including all blood pressure values measured longitudinally from enrollment up to (but not including) the onset of labor) and interactions between treatment groups and gestational age spline terms.

In the placebo group, SBP and DBP remained reasonably constant from 17 to ∼30 weeks gestation, then increased until term (see Figure S1, Supplemental Digital Content 1, http://links.lww.com/HJH/B433). There was little variability across vitamin D groups in the patterns of SBP or DBP change during the second half of pregnancy (Fig. 2). The differences in changes of SBP and DBP between vitamin D groups and placebo across intervals were all small (*P* values >0.10), but the difference for 28 000 IU/week versus placebo was the highest from 30 to 36 weeks [SBP 0.18 (−0.11 to 0.46) and DBP 0.19 (−0.04 to 0.41) mmHg) and was the lowest from 36 weeks to delivery [−0.33 (−0.93 to 0.270) mmHg] (Table 3). Results in models adjusted for serum calcium were similar (data not shown).

**TABLE 3:** Effect of vitamin D supplementation^a^ on changes in maternal blood pressure in intervals across pregnancy, *n* = 1257

	SBP Δ per week (mmHg)^b^			DBP Δ per week (mmHg)^b^		
Gestational age	*β*	95% CI	*P* value^c^	*β*	95% CI	*P* value^c^
24–30 weeks						
Placebo	ref			ref		
4200 IU/week	−0.042	(−0.382 to 0.299)	0.810	−0.038	(−0.311 to 0.235)	0.785
16 800 IU/week	0.018	(−0.327 to 0.362)	0.920	0.034	(−0.242 to 0.310)	0.811
28 000 IU/week	0.130	(−0.166 to 0.426)	0.389	0.103	(−0.134 to 0.341)	0.393
30–36 weeks						
Placebo	ref			ref		
4200 IU/week	0.209	(−0.116 to 0.533)	0.207	0.108	(−0.152 to 0.369)	0.416
16800 IU/week	−0.009	(−0.336 to 0.317)	0.956	0.040	(−0.222 to 0.302)	0.763
28 000 IU/week	0.176	(−0.107 to 0.459)	0.223	0.186	(−0.041 to 0.413)	0.108
>36 weeks						
Placebo	ref			ref		
4200 IU/week	0.098	(−0.573 to 0.769)	0.775	−0.038	(−0.576 to 0.500)	0.890
16 800 IU/week	−0.042	(−0.744 to 0.659)	0.906	0.122	(−0.441 to 0.684)	0.672
28 000 IU/week	−0.326	(−0.926 to 0.274)	0.287	−0.073	(−0.554 to 0.409)	0.767

CI, confidence interval; IU, international unit.

a Vitamin D doses were administered weekly within a randomized controlled trial.

b Mixed-effects models with linear spline knots at 30 and 36 weeks gestation were used to estimate effect of vitamin D on changes in SBP or DBP. Models included spline terms for gestational age; treatment groups and interactions terms between treatment group and gestational age spline terms. Models were fit to test the effect of each vitamin D treatment group on SBP or DBP with reference to placebo. Models were adjusted for baseline SBP or DBP and gestational age at enrollment. Estimates for vitamin D groups are the coefficients for the interaction terms between treatment group and gestational age spline terms, which represent the mean change in SBP or DBP between vitamin D groups compared with placebo at specified timepoints. Interclass correlation coefficient: SBP, 0.399; DBP, 0.413.

c *P* value is for the interaction term between treatment group and gestational age spline terms.

In analyses stratified by low DBP (<60 mmHg and ≥60 mmHg) at enrollment, the increase in blood pressure in the highest dose vitamin D group at 36 weeks remained robust only in the group with DBP at least 60 mmHg; other results were consistent with the overall findings (see Tables S2 and S3, Supplemental Digital Content 1, http://links.lww.com/HJH/B433). When stratifying by vitamin D status at baseline, the increase in SBP and DBP at 36 weeks for the highest dose was not observed in the vitamin D-sufficient group, whereas other findings were similar (see Tables S4 and S5, Supplemental Digital Content 1, http://links.lww.com/HJH/B433).

Our re-examination of the effect of vitamin D on gestational hypertension yielded similar results to the main trial; all relative risk estimates had wide confidence intervals that substantially overlapped the null (see Table S6, Supplemental Digital Content 1, http://links.lww.com/HJH/B433).

Sensitivity analysis performed by limiting to SBP and DBP where differences between two measurements were less than 10 mmHg (*n* = 1193) or term birth (*n* = 1110) resulted in similar findings (see Tables S7, S8, S9 and S10, Supplemental Digital Content 1, http://links.lww.com/HJH/B433).

## DISCUSSION

In a population of women with low vitamin D status and generally low blood pressure, vitamin D supplementation starting mid-pregnancy did not affect SBP or DBP until late gestation, and then only at the highest dose. SBP and DBP were higher by ∼2 mmHg at week 36 in the women receiving the highest dose of vitamin D compared with placebo. Findings were similar across subgroups categorized by DBP at enrollment or baseline vitamin D status; although higher blood pressure from the highest dose was only observed among women having DBP at least 60 mmHg and those with baseline vitamin D deficiency.

The present finding that blood pressure was elevated at 36 weeks gestation in response to vitamin D supplementation differed from three other trials. Asemi *et al.*[17] reported a relative decrease in SBP and DBP over a 9-week period among Iranian women receiving low-dose vitamin D supplementation (400 IU/day) compared with placebo starting at 25 weeks gestation (*n* = 48) [17]. In another Iranian study, by the same group, co-intervention of low-dose vitamin D (200 IU/day) and calcium (500 mg/day) supplements for a period of 9 weeks starting from 25 weeks gestation (*n* = 42) resulted in a decrease in DBP but not SBP among women in the treatment group compared with placebo [18]. Zerofsky *et al.*[19] compared 2000 and 400 IU/day in a small group of women in California (*n* = 57) and reported a nonstatistically significant increase in DBP from the first visit (<20 weeks gestation) to the last visit (35–36 weeks gestation) in the control group but not the treatment group. The trials by Asemi *et al.*[17] and Zerofsky *et al.*[18] were limited by their small sample sizes, low doses of vitamin D, and/or lack of placebo comparator as well as very high baseline vitamin D status [19] and relatively short durations of supplementation [17,18]. Although other trials have reported vitamin D effects on preeclampsia or gestational hypertension, effects on blood pressure dynamics during pregnancy have rarely been described. Most randomized controlled trials start vitamin D intervention after the first trimester, limiting our understanding of the effect on blood pressure during early pregnancy [15]. This is an important constraint, given that blood pressure changes begin very early in gestation, and that spiral artery remodeling and placentation is essentially complete by the end of the first trimester.

Across all treatment groups, average SBP and DBP remained stable from 24 to 30 weeks gestation and then moderately increased from 30 weeks until term. We observed a slight difference in the pattern of blood pressure change from 24 to 30 weeks compared with other studies but the rise in SBP and DBP during the latter half of the third trimester was consistent with other cohorts [11,24]. The mean SBP among women in our study was slightly lower but DBP was similar to other cohorts in other countries [11,24].

We originally hypothesized that vitamin D would attenuate the increase in blood pressure in the third trimester, consistent with numerous observational studies suggesting an association of vitamin D deficiency [or low 25(OH)D] with an increased risk of hypertensive disorders of pregnancy [8]. It has been proposed that vitamin D may favorably regulate blood pressure via its effects on the renin–angiotensin system [25]. However, studies in nonpregnant adults have not substantiated the association between low vitamin D status and hypertension [26]. Moreover, a recent population-based observational study in Sweden demonstrated that 25(OH)D concentrations during the first trimester were positively associated with SBP (though not DBP) trajectory across pregnancy despite a reduced odds of preeclampsia with higher 25(OH)D [10]. These seemingly contradictory findings suggest that an effect of vitamin D on the risk of preeclampsia may be mediated by other pathways (e.g. immunomodulation) unrelated to blood pressure. There is inconsistent evidence in the literature on the potential beneficial effects of vitamin D supplementation on blood pressure and other health conditions even in nonpregnant populations [27].

After finding that high-dose vitamin D increased blood pressure in late pregnancy, we considered the possibility that blood pressure may have been increasing into a normal range, as many women started with low blood pressure. Yet, this hypothesis was not supported by our stratified analyses, which showed instead that the vitamin D effect was stronger for women without low blood pressure at baseline. Given that high-dose vitamin D slightly increases maternal serum calcium concentrations [20], we also considered the possibility that increases in serum calcium may have mediated the increased blood pressure, as suggested by the established association between serum calcium and blood pressure in other cohorts [28,29]. The mechanism may be attributable to effects of calcium on vascular tone, arterial stiffness or catecholamine secretion [30,31]. However, in regression models that controlled for serum calcium at 30 weeks gestation, the effects of vitamin D at 36 weeks were only slightly attenuated. Therefore, rises in serum calcium did not fully explain the effect of vitamin D on blood pressure.

The implications that vitamin D may increase maternal blood pressure during pregnancy are uncertain, particularly given that this trial could not clearly establish whether there were effects on gestational hypertension or other hypertensive morbidities. Although the trial was not designed to capture a diagnosis of preeclampsia, we did examine gestational hypertension, as previously reported [20] and in our current re-analysis. Few cases limited the power to detect an effect, yet there were more cases among those who received vitamin D supplementation compared with placebo. However, given the lack of observed benefit or risk of the high-dose intervention on other birth and infant outcomes in this population [20], the findings related to gestational hypertension do not provide clear evidence of an adverse effect. Our conclusions are consistent with a recent meta-analysis of randomized controlled trials of vitamin D supplementation during pregnancy, which did not find evidence to support a beneficial effect of vitamin D on gestational hypertension (pooled risk ratio of 1.69, 95% CI 0.73 to 3.92) [15]. As very few studies have rigorously examined the effects of vitamin D supplementation during pregnancy on gestational hypertension and preeclampsia, future trials designed and powered to examine hypertensive disorders of pregnancy are needed.

Although this population with very low vitamin D status was ideal to study the effects of vitamin D supplementation, it had an unexpectedly high prevalence of low blood pressure at enrollment. Almost a third of women had DBP less than 60 mmHg at baseline, possibly related to the low average heights and weights of this population compared with cohorts in high-income countries. Other studies examining the impact of vitamin D supplementation on blood pressure in pregnancy should be conducted in settings were vitamin D status is low to examine the potential beneficial effect in other populations.

Strengths of this study were the randomized, placebo-controlled, and dose-ranging design, large sample size, and repeated measurements of blood pressure across pregnancy. Women in the study were generally healthy at baseline, and we are not aware of any pregnancies because of assisted reproductive technologies. A notable limitation was that the intervention was initiated in the second trimester, and earlier gestational effects of vitamin D may differ from those we observed in the second half of pregnancy. Further, although blood pressure was a planned measurement, the objective of the current study was not a prespecified analysis. Standardized diagnosis of preeclampsia was not implemented as a routine study procedure. Preeclampsia would have been rare in this population and the study was underpowered to examine this outcome. Women with pregnancy complications were referred for treatment by nonstudy clinicians, so treatment for high blood pressure was not standardized as a part of study protocols neither were details of antihypertensive treatment systematically captured. Since this study is a randomized controlled trial with a masked intervention, we assumed that physicians’ decisions regarding treatment of hypertension were similar across groups.

More frequent blood pressure measurement may have improved the precision of our estimates. And, a larger sample size would have enabled more robust inferences with respect to effects on gestational hypertension. The unique characteristics of the cohort, including the high prevalence of vitamin D deficiency and low DBP and short stature, may limit generalizability to populations where women have comparatively higher stature, blood pressure and vitamin D status.

In conclusion, our findings did not support the hypothesis that vitamin D lowers blood pressure across the second half of pregnancy. Rather, we found that the highest vitamin D dose (equivalent to ∼4000 IU/day) compared with placebo resulted in a higher blood pressure at 36 weeks gestation, probably resulting from a slightly higher rate of increase from 30 to 36 weeks. The mechanism and significance of this observed effect remains unexplained. Additional randomized controlled trials focused on blood pressure dynamics and hypertensive disorders of pregnancy as primary outcomes are needed to further guide public health recommendations for vitamin D supplementation during pregnancy. Of particular importance will be trials beginning in early pregnancy or periconception, when the earliest physiologic changes of pregnancy are occurring that may set a trajectory toward later hypertensive disorders.

## ACKNOWLEDGEMENTS

We would like to thank Huma Qamar for collating the datasets used in this study. We also thank all the MDIG staff at icddr,b, Centre for Global Child Health, Hospital for Sick Children, Toronto and the participants of the study.

The MDIG trial was funded by the Bill and Melinda Gates Foundation (OPP1066764).

Trial Registration: ClinicalTrials.gov (NCT01924013)

### Conflicts of interest

There are no conflicts of interest.

## Supplementary Material

Supplemental Digital Content
