# Supplementary material for: Effect of vitamin D supplementation during pregnancy on mid-to-late gestational blood pressure in a randomized controlled trial in Bangladesh
Source: J Hypertens. 2020 Aug 10;39(1):135–42. doi: 10.1097/HJH.0000000000002609 (PMC7752208; doi:10.1097/HJH.0000000000002609)
Supplement: Supplemental Digital Content [file jhype-39-135-s001.docx]

| **Table S1. Maternal blood pressure across pregnancy by treatment group.**^1^ | | | | | | | | | |
| --- | --- | --- | --- | --- | --- | --- | --- | --- | --- |
|  | **Vitamin D Treatment Group**^2^ | | | | | | | | |
|  | **SBP (mm Hg)** | | | |  | **DBP (mm Hg)** | | | |
|  | **Placebo** | **4200 IU/wk** | **16800 IU/wk** | **28000 IU/wk** |  | **Placebo** | **4200 IU/wk** | **16800 IU/wk** | **28000 IU/wk** |
| **Gestational Age** |  |  |  |  |  |  |  |  |  |
| 23 weeks | 100.8 $\pm$ 9.5 | 101.8 $\pm$ 9.1 | 102.2 $\pm$ 10.0 | 101.3 $\pm$ 8.9 |  | 64.6 $\pm$7.8 | 65.8 $\pm$ 6.2 | 65.1 $\pm$ 8.7 | 64.7 $\pm$ 7.0 |
| N | 75 | 80 | 68 | 167 |  | 75 | 80 | 68 | 167 |
| 24 weeks | 101.2 $\pm$ 10.5 | 101.1 $\pm$ 9.7 | 101.4 $\pm$10.0 | 102.7 $\pm$ 9.8 |  | 64.9 $\pm$ 8.2 | 65.0 $\pm$ 7.3 | 64.8 $\pm$ 8.0 | 65.7 $\pm$ 8.3 |
| N | 119 | 117 | 125 | 231 |  | 119 | 117 | 125 | 231 |
| 29 weeks | 96.8 $\pm$ 9.1 | 97.0 $\pm$ 8.9 | 98.2 $\pm$ 9.4 | 98.7 $\pm$ 9.9 |  | 61.7 $\pm$ 7.3 | 62.4 $\pm$ 7.0 | 63.0 $\pm$ 8.2 | 62.8 $\pm$6.1 |
| N | 88 | 76 | 83 | 189 |  | 88 | 76 | 83 | 189 |
| 30 weeks | 98.4 $\pm$ 10.6 | 98.6 $\pm$ 9.7 | 99.1 $\pm$ 11.0 | 99.2 $\pm$ 9.9 |  | 62.9 $\pm$ 7.7 | 63.0 $\pm$ 7.6 | 62.9 $\pm$ 7.6 | 63.2 $\pm$ 8.0 |
| N | 134 | 145 | 134 | 268 |  | 134 | 145 | 134 | 268 |
| 35 weeks | 103.7 $\pm$ 10.0 | 103.9 $\pm$ 8.3 | 105.6 $\pm$ 11.1 | 105.2 $\pm$ 10.3 |  | 67.8 $\pm$ 8.1 | 69.0 $\pm$ 7.0 | 69.5 $\pm$ 8.8 | 69.7 $\pm$8.6 |
| N | 81 | 80 | 74 | 162 |  | 81 | 80 | 74 | 162 |
| 36 weeks | 104.7 $\pm$ 10.0 | 105.4 $\pm$ 10.1 | 105.0 $\pm$ 10.8 | 106.4 $\pm$ 10.7 |  | 68.9 $\pm$ 8.1 | 69.7 $\pm$8.7 | 69.0 $\pm$ 9.1 | 70.1 $\pm$ 8.7 |
| N | 189 | 184 | 198 | 362 |  | 189 | 184 | 198 | 362 |
| 37 weeks | 105.5 $\pm$ 11.3 | 107.2 $\pm$ 11.2 | 105.7 $\pm$ 9.8 | 108.0 $\pm$ 12.7 |  | 69.5 $\pm$ 8.2 | 70.3 $\pm$ 9.3 | 69.7 $\pm$ 8.1 | 71.3 $\pm$9.3 |
| N | 153 | 182 | 173 | 341 |  | 153 | 182 | 173 | 341 |
| 38 weeks | 105.8 $\pm$ 10.3 | 106.8 $\pm$ 11.4 | 106.2 $\pm$ 11.0 | 108.4 $\pm$ 12.4 |  | 70.1 $\pm$ 8.4 | 70.2 $\pm$ 8.6 | 70.5 $\pm$ 8.8 | 71.9 $\pm$9.3 |
| N | 135 | 140 | 134 | 277 |  | 135 | 140 | 134 | 277 |
| 39 weeks | 108.4 $\pm$ 10.0 | 108.8 $\pm$ 12.2 | 107.8 $\pm$ 10.8 | 108.6 $\pm$ 12.0 |  | 71.7 $\pm$ 7.2 | 72.2 $\pm$ 9.3 | 71.4 $\pm$9.1 | 72.5 $\pm$9.6 |
| N | 83 | 80 | 83 | 176 |  | 83 | 80 | 83 | 175 |
| 40 weeks | 112.1 $\pm$ 11.3 | 110.9 $\pm$ 15.0 | 109.5 $\pm$ 10.3 | 109.5 $\pm$ 13.1 |  | 76.2 $\pm$ 8.1 | 74.0 $\pm$ 11.4 | 74.4 $\pm$ 8.4 | 72.8 $\pm$10.1 |
| N | 30 | 44 | 26 | 70 |  | 30 | 44 | 26 | 70 |

DBP, diastolic blood pressure; IU, international unit; SBP, systolic blood pressure

^1^Data are mean $\pm$SD.

^2^Vitamin D doses were administered weekly within a randomized controlled trial.

| **Table S2. Effect of vitamin D supplementation**^1^ **on maternal blood pressure at 24, 30 and 36 weeks gestation, stratified by maternal low blood pressure at enrollment (n=1257).** | | | | | | |
| --- | --- | --- | --- | --- | --- | --- |
|  | **Mean (95% CI)** |  | **Mean difference (95% CI)**^2,3^ | | |  |
|  | **Placebo** |  | **4200 IU/wk**^3^ | **16800 IU/wk**^3^ | **28000 IU/wk**^3^ | **P-Value**^4^ |
| **DBP  <60 mm Hg**^5^  **(n=344)** |  |  |  |  |  |  |
| SBP (mm Hg) |  |  |  |  |  |  |
| 24 weeks | 97.0 (94.7, 99.2) |  | 0.16 (-3.12, 3.44) | 0.95 (-2.33, 4.23) | 1.15 (-1.62, 3.93) | 0.820 |
| 30 weeks | 92.6 (90.3, 94.9) |  | 1.10 (-2.20, 4.42) | -0.16 (-3.53, 3.21) | 1.70 (-1.10 4.48) | 0.519 |
| 36 weeks | 100.9 (99.0, 103.1) |  | -0.71 (-3.73, 2.29) | 0.11 (-2.90, 3.12) | 1.78 (-0.80, 4.37) | 0.199 |
| DBP (mm Hg) |  |  |  |  |  |  |
| 24 weeks | 62.3 (60.4, 64.2) |  | -0.41 (-3.17, 2.34) | -1.71 (-4.47, 1.04) | -0.80 (-3.13, 1.53) | 0.659 |
| 30 weeks | 59.0 (57.0, 60.9) |  | -0.50 (-3.29, 2.28) | -2.22 (-5.05, 0.61) | 0.31 (-2.02, 2.67) | 0.238 |
| 36 weeks | 66.0 (64.2, 68.0) |  | -1.24 (-3.77, 1.28) | -1.80 (-4.31, 0.72) | 0.66 (-1.50, 2.82) | 0.091 |
| **DBP  ≥60 mm Hg**  **(n=913)** |  |  |  |  |  |  |
| SBP (mm Hg) |  |  |  |  |  |  |
| 24 weeks | 102.5 (101.0, 104.0) |  | 0.76 (-1.35, 2.87) | 0.31 (-1.80, 2.44) | 0.29 (-1.56, 2.13) | 0.916 |
| 30 weeks | 99.7 (98.1, 101.2) |  | -0.03 (-2.22, 2.15) | 0.75 (-1.44, 2.94) | 1.11 (-0.80, 3.03) | 0.550 |
| 36 weeks | 105.7 (104.3, 107.0)^a^ |  | 2.44 (0.54, 4.34)^b^ | 0.69 (-1.21, 2.60) | 2.58 (0.91, 4.25)^b^ | 0.006 |
| DBP (mm Hg) |  |  |  |  |  |  |
| 24 weeks | 65.9 (64.7, 67.1) |  | 0.63 (-1.06, 2.32) | 0.23 (-1.47, 1.93) | 0.47 (-1.00, 1.95) | 0.883 |
| 30 weeks | 63.8 (62.5, 65.0) |  | 0.28 (-1.46, 2.04) | 0.64 (-1.11, 2.39) | 0.90 (-0.63, 2.43) | 0.673 |
| 36 weeks | 69.7 (68.6, 70.7)^c^ |  | 1.47 (-0.05, 2.99) | 0.87 (-0.66, 2.40) | 2.32 (0.98, 3.67)^d^ | 0.006 |

CI, confidence interval; DBP, diastolic blood pressure; IU, international unit; SBP, systolic blood pressure

^1^Vitamin D doses were administered weekly within a randomized controlled trial.

^2^Mixed-effects models with linear spline knots at 30, and 36 weeks gestation were used to estimate effect of vitamin D on changes in SBP or DBP. Models included spline terms for gestational age; treatment group; and interaction terms between treatment group and gestational age spline terms. Models were fit to test the effect of each vitamin D treatment group on SBP or DBP with reference to placebo and stratified by maternal low blood pressure at enrollment. Models were adjusted for baseline SBP or DBP and gestational age at enrollment. Marginal effects of treatment group were examined on SBP or DBP at 24, 30, and 36 weeks gestation.

^3^Values represent mean difference in SBP or DBP for each vitamin D group compared to placebo.

^4^P-value represents overall difference in means across treatment groups.

^5^Defined as DBP <60 mm Hg at baseline when two sets of DBP measurements within a visit were below the cut-off; often used as a cut-point for low blood pressure.

^ab^SBP was different between vitamin D group compared to placebo; estimated using the contrast of margins (4200IU/wk, p=0.01; 28000 IU/wk, p=0.002).

^cd^DBP was different between vitamin D group compared to placebo; estimated using the contrast of margins (p<0.001).

| **Table S3. Effect of vitamin D supplementation**^1^ **on changes in maternal blood pressure in intervals across pregnancy, stratified by maternal low blood pressure at enrollment (n=1257).** | | | | | | | |
| --- | --- | --- | --- | --- | --- | --- | --- |
|  | **DBP  <60 mm Hg**^2,3^  **(n=344)** | | |  | **DBP  ≥60 mm Hg**^2^  **(n=913)** | | |
|  | **β** | **95 % CI** | **P- Value**^4^ |  | **β** | **95 % CI** | **P- Value**^4^ |
| **SBP (mm Hg)** |  |  |  |  |  |  |  |
| **24 to 30 weeks** |  |  |  |  |  |  |  |
| Placebo | ref |  |  |  | ref |  |  |
| 4200 IU/wk | 0.159 | (-0.460, 0.778) | 0.616 |  | -0.132 | (-0.539, 0.273) | 0.522 |
| 16800 IU/wk | -0.185 | (-0.807, 0.437) | 0.560 |  | 0.072 | (-0.339, 0.483) | 0.730 |
| 28000 IU/wk | 0.090 | (-0.431, 0.610) | 0.736 |  | 0.138 | (-0.218, 0.495) | 0.447 |
| **30 to 36 weeks** |  |  |  |  |  |  |  |
| Placebo | ref |  |  |  | ref |  |  |
| 4200 IU/wk | -0.304 | (-0.899, 0.290) | 0.316 |  | 0.414 | (0.028, 0.800) | 0.036 |
| 16800 IU/wk | 0.046 | (-0.549, 0.641) | 0.878 |  | -0.010 | (-0.400, 0.378) | 0.957 |
| 28000 IU/wk | 0.015 | (-0.487, 0.519) | 0.951 |  | 0.244 | (-0.096, 0.584) | 0.160 |
| **>36 weeks** |  |  |  |  |  |  |  |
| Placebo | ref |  |  |  | ref |  |  |
| 4200 IU/wk | -0.232 | (-1.592, 1.127) | 0.738 |  | 0.149 | (-0.623, 0.922) | 0.705 |
| 16800 IU/wk | -0.999 | (-2.34, 0.348) | 0.146 |  | 0.255 | (-0.571, 1.081) | 0.545 |
| 28000 IU/wk | -0.780 | (-1.979, 0.419) | 0.202 |  | -0.244 | (-0.940, 0.451) | 0.491 |
| **DBP (mm Hg)** |  |  |  |  |  |  |  |
| **24 to 30 weeks** |  |  |  |  |  |  |  |
| Placebo | ref |  |  |  | ref |  |  |
| 4200 IU/wk | -0.014 | (-0.539 0.510) | 0.957 |  | -0.057 | (-0.377, 0.262) | 0.723 |
| 16800 IU/wk | -0.085 | (-0.612, 0.442) | 0.752 |  | 0.068 | (-0.256, 0.391) | 0.681 |
| 28000 IU/wk | 0.186 | (-0.255, 0.628) | 0.410 |  | 0.070 | (-0.209, 0.351) | 0.621 |
| **30 to 36 weeks** |  |  |  |  |  |  |  |
| Placebo | ref |  |  |  | ref |  |  |
| 4200 IU/wk | -0.124 | (-0.629, 0.380) | 0.628 |  | 0.197 | (-0.107, 0.501) | 0.203 |
| 16800 IU/wk | 0.071 | (-0.433, 0.576) | 0.782 |  | 0.039 | (-0.268, 0.345) | 0.804 |
| 28000 IU/wk | 0.056 | (-0.370, 0.483) | 0.796 |  | 0.237 | (-0.031, 0.504) | 0.083 |
| **>36 weeks** |  |  |  |  |  |  |  |
| Placebo | ref |  |  |  | ref |  |  |
| 4200 IU/wk | 0.027 | (-1.126, 1.179) | 0.964 |  | -0.083 | (-0.692, 0.525) | 0.788 |
| 16800 IU/wk | -0.136 | (-1.279, 1.007) | 0.816 |  | 0.185 | (-0.465, 0.836) | 0.577 |
| 28000 IU/wk | -0.059 | (-1.075, 0.957) | 0.909 |  | -0.119 | (-0.668, 0.429) | 0.669 |

CI, confidence interval; DBP, diastolic blood pressure; IU, international unit; SBP, systolic blood pressure.

^1^Vitamin D doses were administered weekly within a randomized controlled trial.

^2^Mixed-effects models with linear spline knots at 30, and 36 weeks gestation were used to estimate effect of vitamin D on changes in SBP or DBP. Models included spline terms for gestational age; treatment groups; and interactions terms between treatment group and gestational age spline terms. Models were fit to test the effect of each vitamin D treatment group on SBP or DBP with reference to placebo and stratified by maternal low blood pressure at enrollment. Models were adjusted for baseline SBP or DBP and gestational age at enrollment. Estimates for vitamin D groups are the coefficients for the interaction terms between treatment group and gestational age spline terms which represent the mean change in SBP or DBP between vitamin D groups compared to placebo at specified timepoints.

^3^Defined as DBP <60 mm Hg at baseline when two sets of DBP measurements within a visit were below the cut-off; often used as a cut-point for low blood pressure.

^4^P-value is for the interaction term between treatment group and gestational age spline terms.

| **Table S4. Effect of vitamin D supplementation**^1^ **on maternal blood pressure at 24, 30 and 36 weeks gestation, stratified by baseline vitamin D status.** | | | | | | |
| --- | --- | --- | --- | --- | --- | --- |
|  | **Mean (95% CI)** |  | **Mean difference (95% CI)**^2,3^ | | |  |
|  | **Placebo** |  | **4200 IU/wk**^3^ | **16800 IU/wk**^3^ | **28000 IU/wk**^3^ | **P-Value**^4^ |
| **25(OH)D**  **<30 nmol/L**^5^  **(n=800)** |  |  |  |  |  |  |
| SBP (mm Hg) |  |  |  |  |  |  |
| 24 weeks | 100.9 (99.3, 102.6) |  | 0.40 (-1.84, 2.66) | 0.29 (-1.98, 2.55) | -0.04 (-1.96, 1.87) | 0.964 |
| 30 weeks | 97.2 (95.6, 99.0) |  | 0.71 (-1.61, 3.04) | 0.29 (-2.08, 2.66) | 1.52 (-0.48, 3.53) | 0.412 |
| 36 weeks | 104.1 (102.6, 105.6)^a^ |  | 1.77 (-0.27, 3.80) | 0.91 (-1.12, 2.96) | 2.61 (0.85, 4.37)^b^ | 0.022 |
| DBP (mm Hg) |  |  |  |  |  |  |
| 24 weeks | 64.8 (63.5, 66.1) |  | 0.15 (-1.68, 1.99) | -0.71 (-2.57, 1.13) | -0.44 (-2.01, 1.12) | 0.760 |
| 30 weeks | 61.7 (60.3, 63.0) |  | 0.26 (-1.63, 2.15) | -0.02 (-1.96, 1.90) | 1.29 (-0.34, 2.93) | 0.257 |
| 36 weeks | 68.4 (67.2, 70.0)^c^ |  | 1.01 (-0.654, 2.68) | 0.29 (-1.38, 1.97) | 1.83 (0.39, 3.28)^d^ | 0.042 |
| **25(OH)D**  **≥30 nmol/L**  **(n=451)** |  |  |  |  |  |  |
| SBP (mm Hg) |  |  |  |  |  |  |
| 24 weeks | 100.1 (99.1, 103.2) |  | 0.80 (-2.10, 3.71) | 0.81 (-2.10, 3.72) | 1.44 (-1.16, 4.03) | 0.754 |
| 30 weeks | 98.1 (96.0, 100.2) |  | 0.01 (-2.96, 2.99) | 1.47 (-1.46, 4.40) | 0.99 (-1.62, 3.60) | 0.685 |
| 36 weeks | 104.9 (103.0, 107.0) |  | 1.34 (-1.28, 3.98) | 0.13 (-2.49, 2.77) | 1.64 (-0.70, 3.98) | 0.418 |
| DBP (mm Hg) |  |  |  |  |  |  |
| 24 weeks | 65.1 (63.4, 66.8) |  | 0.47 (-1.86, 2.80) | 0.30 (-2.02, 2.64) | 1.28 (-0.80, 3.36) | 0.618 |
| 30 weeks | 63.7 (62.0, 65.3) |  | -0.11 (-2.49, 2.27) | -0.08 (-2.43, 2.27) | -0.17 (-2.26, 1.92) | 0.999 |
| 36 weeks | 69.2 (67.7, 70.7) |  | 0.20 (-1.90, 2.29) | -0.03 (-2.13, 2.06) | 1.78 (-0.08, 3.65) | 0.120 |

CI, confidence interval; DBP, diastolic blood pressure; IU, international unit; SBP, systolic blood pressure; 25(OH)D, 25-hydroxyvitamin D

^1^Vitamin D doses were administered weekly within a randomized controlled trial.

^2^Mixed-effects models with linear spline knots at 30, and 36 weeks gestation were used to estimate effect of vitamin D on changes in SBP or DBP. Models included spline terms for gestational age; treatment group; and interaction terms between treatment group and gestational age spline terms. Models were fit to test the effect of each vitamin D treatment group on SBP or DBP with reference to placebo and stratified by baseline vitamin D status. Models were adjusted for baseline SBP or DBP and gestational age at enrollment. Marginal effects of treatment group were examined on SBP or DBP at 24, 30, and 36 weeks gestation.

^3^Values represent mean difference in SBP or DBP for each vitamin D group compared to placebo.

^4^ P-value represents overall difference in means across treatment groups.

^5^Vitamin D deficiency was defined as a concentration of 25(OH)D <30 nmol/L.

^ab^SBP was different between vitamin D group compared to placebo; estimated using the contrast of margins (p=0.003).

^cd^DBP was different between vitamin D group compared to placebo; estimated using the contrast of margins (p=0.012).

| **Table S5. Effect of vitamin D supplementation**^1^ **on changes in maternal blood pressure in intervals across pregnancy, stratified by baseline vitamin D status.** | | | | | | | |
| --- | --- | --- | --- | --- | --- | --- | --- |
|  | **25 (OH) D**  **<30 nmol/L**^2,3^  **(n=800)** | | |  | **25 (OH) D**  **≥30 nmol/L**^2^  **(n=451)** | | |
|  | **β** | **95 % CI** | **P- Value**^3^ |  | **β** | **95 % CI** | **P- Value**^3^ |
| **SBP (mm Hg)** |  |  |  |  |  |  |  |
| **24 to 30 weeks** |  |  |  |  |  |  |  |
| Placebo | ref |  |  |  | ref |  |  |
| 4200 IU/wk | 0.051 | (-0.382, 0.485) | 0.816 |  | -0.131 | (-0.680, 0.417) | 0.638 |
| 16800 IU/wk | 0.000 | (-0.442, 0.443) | 0.999 |  | 0.109 | (-0.438, 0.655) | 0.695 |
| 28000 IU/wk | 0.261 | (-0.111, 0.636) | 0.170 |  | -0.076 | (-0.561, 0.410) | 0.760 |
| **30 to 36 weeks** |  |  |  |  |  |  |  |
| Placebo | ref |  |  |  | ref |  |  |
| 4200 IU/wk | 0.174 | (-0.240, 0.590) | 0.409 |  | 0.221 | (-0.298, 0.740) | 0.404 |
| 16800 IU/wk | 0.104 | (-0.318, 0.526) | 0.628 |  | -0.221 | (-0.736, 0.292) | 0.399 |
| 28000 IU/wk | 0.180 | (-0.180, 0.541) | 0.327 |  | 0.108 | (-0.348, 0.567) | 0.641 |
| **>36 weeks** |  |  |  |  |  |  |  |
| Placebo | ref |  |  |  | ref |  |  |
| 4200 IU/wk | 0.396 | (-0.469, 1.260) | 0.370 |  | -0.307 | (-1.366, 0.749) | 0.568 |
| 16800 IU/wk | 0.100 | (-0.800, 1.00) | 0.827 |  | -0.206 | (-1.317, 0.906) | 0.717 |
| 28000 IU/wk | -0.060 | (-0.827, 0.706) | 0.878 |  | -0.663 | (-1.630, 0.303) | 0.179 |
| **DBP (mm Hg)** |  |  |  |  |  |  |  |
| **24 to 30 weeks** |  |  |  |  |  |  |  |
| Placebo | ref |  |  |  | ref |  |  |
| 4200 IU/wk | 0.017 | (-0.327, 0.361) | 0.922 |  | -0.098 | (-0.544, 0.349) | 0.668 |
| 16800 IU/wk | 0.114 | (-0.237, 0.467) | 0.522 |  | -0.065 | (-0.510, 0.380) | 0.775 |
| 28000 IU/wk | 0.290 | (-0.007, 0.586) | 0.056 |  | -0.241 | (-0.638, 0.154) | 0.232 |
| **30 to 36 weeks** |  |  |  |  |  |  |  |
| Placebo | ref |  |  |  | ref |  |  |
| 4200 IU/wk | 0.126 | (-0.204, 0.456) | 0.454 |  | 0.052 | (-0.370, 0.476) | 0.807 |
| 16800 IU/wk | 0.0531 | (-0.282, 0.388) | 0.756 |  | 0.008 | (-0.411, 0.427) | 0.970 |
| 28000 IU/wk | 0.0914 | (-0.195, 0.378) | 0.532 |  | 0.325 | (-0.047, 0.698) | 0.087 |
| **>36 weeks** |  |  |  |  |  |  |  |
| Placebo | ref |  |  |  | ref |  |  |
| 4200 IU/wk | 0.048 | (-0.639, 0.735) | 0.891 |  | -0.113 | (-0.974, 0.748) | 0.797 |
| 16800 IU/wk | 0.304 | (-0.412, 1.021) | 0.405 |  | -0.130 | (-1.035, 0.774) | 0.778 |
| 28000 IU/wk | 0.181 | (-0.429, 0.791) | 0.560 |  | -0.454 | (-1.242, 0.332) | 0.258 |

CI, confidence interval; DBP, diastolic blood pressure; IU, international unit; SBP, systolic blood pressure; 25(OH)D, 25-hydroxyvitamin D

^1^Vitamin D doses were administered weekly within a randomized controlled trial.

^2^Mixed-effects models with linear spline knots at 30, and 36 weeks gestation were used to estimate effect of vitamin D on changes in SBP or DBP. Models included spline terms for gestational age; treatment groups; and interactions terms between treatment group and gestational age spline terms. Models were fit to test the effect of each vitamin D treatment group on SBP or DBP with reference to placebo and stratified by baseline vitamin D status. Models were adjusted for baseline SBP or DBP and gestational age at enrollment. Estimates for vitamin D groups are the coefficients for the interaction terms between treatment group and gestational age spline terms which represent the mean change in SBP or DBP between vitamin D groups compared to placebo at specified timepoints.

^3^Vitamin D deficiency was defined as a concentration of 25(OH)D <30 nmol/L.

^4^P-value is for the interaction term between treatment group and gestational age spline terms.

| **Table S6. Effect of vitamin D on risk of gestational hypertension (n=1257).**^1^ | | | | | |
| --- | --- | --- | --- | --- | --- |
| **Vitamin D Treatment Group**^2^ | **Cases/Total**^3^ | **Incidence per 1000 live births** | **RR**^4^ | **95% CI** | **P-Value** |
| Placebo | 9/250 | 36.0 | ref |  |  |
| 4200 IU/wk | 13/252 | 51.6 | 1.43 | (0.62, 3.29) | 0.397 |
| 16800 IU/wk | 12/251 | 48.0 | 1.33 | (0.57, 3.10) | 0.511 |
| 28000 IU/wk | 29/504 | 57.5 | 1.60 | (0.77, 3.33) | 0.210 |

CI, confidence interval; IU, international unit; RR, risk ratio.

^1^Analysis by treatment group, including cases defined by severe adverse events, is reported in the main MDIG trial paper table S27 [1].

^2^Vitamin D doses were administered weekly within a randomized controlled trial.

^3^Denominators represents the total number of women in each treatment group. Overall cumulative incidence of gestational hypertension was 5%.

^4^Log-binomial regression models were used to estimate risk ratios. Models included a dichotomous outcome for gestational hypertension and vitamin D treatment groups. Gestational hypertension was classified as systolic blood pressure (SBP) ≥140 mm Hg and/or diastolic blood pressure (DBP) ≥90 mm Hg at any point after enrollment (17-24 weeks) when two sets of SBP and/or DBP measurements within a visit were above the cut-off. If only one set of SBP and/or DBP measurements were available, then the single set was used to define gestational hypertension.

| **Table S7. Effect of vitamin D supplementation**^1^ **on maternal blood pressure at 24, 30 and 36 weeks gestation by limiting to SBP and DBP where differences between two measurements were <10 mm Hg (n=1193).** | | | | | | |
| --- | --- | --- | --- | --- | --- | --- |
|  | **Mean (95% CI)** |  | **Mean difference (95% CI)**^2,3^ | | |  |
|  |  |  |  |  |  |  |
| **SBP (mm Hg)** | **Placebo** |  | **4200 IU/wk**^3^ | **16800 IU/wk**^3^ | **28000 IU/wk**^3^ | **P-Value**^4^ |
| 24 weeks | 101.0 (99.7, 102.3) |  | 0.49 (-1.35, 2.33) | 0.26 (-1.58, 2.10) | 0.59 (-1.01, 2.19) | 0.898 |
| 30 weeks | 97.9 (96.5, 99.3) |  | 0.12 (-1.78, 2.02) | 0.00 (-1.90, 1.91) | 0.91 (-0.74, 2.56) | 0.575 |
| 36 weeks | 104.4 (103.2, 105.6)^a^ |  | 1.68 (0.02, 3.34) | 0.32 (-1.34, 1.98) | 2.09 (0.63, 3.54)^b^ | 0.012 |
| **DBP (mm Hg)** |  |  |  |  |  |  |
| 24 weeks | 64.8 (63.7, 65.9) |  | 0.33 (-1.17, 1.82) | -0.22 (-1.71, 1.27) | 0.26 (-1.04, 1.56) | 0.860 |
| 30 weeks | 62.4 (61.3, 63.5) |  | 0.05 (-1.49, 1.59) | -0.18 (-1.72, 1.37) | 0.68 (-0.66, 2.01) | 0.538 |
| 36 weeks | 68.6 (67.6, 69.6)^c^ |  | 1.04 (-0.31, 2.40) | -0.04 (-1.39, 1.32) | 1.68 (0.49, 2.87)^d^ | 0.007 |

CI, confidence interval; DBP, diastolic blood pressure; IU, international unit; SBP, systolic blood pressure.

^1^Vitamin D doses were administered weekly within a randomized controlled trial.

^2^Mixed-effects models with linear spline knots at 30 and 36 weeks gestation were used to estimate effect of vitamin D on changes in SBP or DBP. Models included spline terms for gestational age; treatment group; and interaction terms between treatment group and gestational age spline terms. Models were fit to test the effect of each vitamin D treatment group on SBP or DBP with reference to placebo. Models were adjusted for baseline SBP or DBP and gestational age at enrollment. Marginal effects of treatment group were examined on SBP or DBP at 24, 30, and 36 weeks gestation.

^3^Values represent mean difference in SBP or DBP for each vitamin D group compared to placebo.

^4^P-value represents overall difference in means across treatment groups.

^ab^SBP was different between vitamin D group compared to placebo; estimated using the contrast of margins (p<0.01)

^cd^DBP was different between vitamin D group compared to placebo; estimated using the contrast of margins (p<0.01)

| **Table S8.** **Effect of vitamin D supplementation**^1^**on changes in maternal blood pressure in intervals across pregnancy by limiting to SBP and DBP where differences between two measurements were <10 mm Hg (n=1193).** | | | | | | | |
| --- | --- | --- | --- | --- | --- | --- | --- |
|  | **SBP Δ per week (mm Hg)**^2^ | | |  | **DBP Δ per week (mm Hg)**^2^ | | |
| **Gestational Age** | **β** | **95% CI** | **P-Value**^3^ |  | **β** | **95% CI** | **P-Value**^3^ |
| **24 to 30 weeks** |  |  |  |  |  |  |  |
| Placebo | ref |  |  |  | ref |  |  |
| 4200 IU/wk | -0.061 | (-0.422, 0.299) | 0.738 |  | -0.046 | (-0.334, 0.242) | 0.752 |
| 16800 IU/wk | -0.043 | (-0.406, 0.320) | 0.816 |  | 0.008 | (-0.282, 0.297) | 0.959 |
| 28000 IU/wk | 0.053 | (-0.261, 0.366) | 0.742 |  | 0.070 | (-0.180, 0.320) | 0.586 |
| **30 to 36 weeks** |  |  |  |  |  |  |  |
| Placebo | ref |  |  |  | ref |  |  |
| 4200 IU/wk | 0.260 | (-0.085, 0.604) | 0.140 |  | 0.166 | (-0.109, 0.441) | 0.238 |
| 16800 IU/wk | 0.054 | (-0.292, 0.400) | 0.760 |  | 0.024 | (-0.253, 0.300) | 0.867 |
| 28000 IU/wk | 0.196 | (-0.105, 0.497) | 0.201 |  | 0.168 | (-0.073, 0.408) | 0.172 |
| **>36 weeks** |  |  |  |  |  |  |  |
| Placebo | ref |  |  |  | ref |  |  |
| 4200 IU/wk | -0.102 | (-0.819, 0.614) | 0.779 |  | -0.195 | (-0.768, 0.378) | 0.505 |
| 16800 IU/wk | -0.064 | (-0.804, 0.676) | 0.865 |  | 0.140 | (-0.452, 0.731) | 0.644 |
| 28000 IU/wk | -0.355 | (-0.996, 0.285) | 0.277 |  | -0.056 | (-0.568, 0.457) | 0.831 |

CI, confidence interval; DBP, diastolic blood pressure; IU, international unit; SBP, systolic blood pressure.

^1^Vitamin D doses were administered weekly within a randomized controlled trial.

^2^Mixed-effects models with linear spline knots at 30, and 36 weeks gestation were used to estimate effect of vitamin D on changes in SBP or DBP. Models included spline terms for gestational age; treatment groups; and interactions terms between treatment group and gestational age spline terms. Models were fit to test the effect of each vitamin D treatment group on SBP or DBP with reference to placebo. Models were adjusted for baseline SBP or DBP and gestational age at enrollment. Estimates for vitamin D groups are the coefficients for the interaction terms between treatment group and gestational age spline terms which represent the mean change in SBP or DBP between vitamin D groups compared to placebo at specified timepoints.

^3^P-value is for the interaction term between treatment group and gestational age spline terms.

| **Table S9. Effect of vitamin D supplementation**^1^ **on maternal blood pressure at 24, 30 and 36 weeks gestation by limiting to term births (n=1110).** | | | | | | |
| --- | --- | --- | --- | --- | --- | --- |
|  | **Mean (95% CI)** |  | **Mean difference (95% CI)**^2,3^ | | |  |
|  |  |  |  |  |  |  |
| **SBP (mm Hg)** | **Placebo** |  | **4200 IU/wk**^3^ | **16800 IU/wk**^3^ | **28000 IU/wk**^3^ | **P-Value**^4^ |
| 24 weeks | 101.0 (99.6, 102.3) |  | 0.78 (-1.07, 2.63) | 0.42 (-1.45, 2.30) | 0.43 (-1.18, 2.05) | 0.878 |
| 30 weeks | 97.8 (96.4, 99.1) |  | 0.71 (-1.19, 2.61) | 0.26 (-1.68, 2.19) | 0.66 (-0.98, 2.34) | 0.837 |
| 36 weeks | 104.5 (103.3, 105.7)^a^ |  | 1.44 (-0.19, 3.07) | 0.17 (-1.47, 1.80) | 1.76 (0.34, 3.18)^b^ | 0.033 |
| **DBP (mm Hg)** |  |  |  |  |  |  |
| 24 weeks | 64.8 (63.7, 65.9) |  | 0.73 (-0.78, 2.25) | -0.13 (-1.67, 1.40) | -0.01 (-1.33, 1.31) | 0.646 |
| 30 weeks | 62.5 (61.4, 63.6) |  | 0.34 (-1.22, 1.90) | -0.49 (-2.07, 1.09) | 0.34 (-1.02, 1.70) | 0.647 |
| 36 weeks | 68.7 (67.8, 69.7)^c^ |  | 0.85 (-0.49, 2.19) | -0.14 (-1.49, 1.20) | 1.47 (0.30, 2.64)^d^ | 0.017 |

CI, confidence interval; DBP, diastolic blood pressure; IU, international unit; SBP, systolic blood pressure.

^1^Vitamin D doses were administered weekly within a randomized controlled trial.

^2^Mixed-effects models with linear spline knots at 30 and 36 weeks gestation were used to estimate effect of vitamin D on changes in SBP or DBP. Models included spline terms for gestational age; treatment group; and interaction terms between treatment group and gestational age spline terms. Models were fit to test the effect of each vitamin D treatment group on SBP or DBP with reference to placebo. Models were adjusted for baseline SBP or DBP and gestational age at enrollment. Marginal effects of treatment group were examined on SBP or DBP at 24, 30, and 36 weeks gestation.

^3^Values represent mean difference in SBP or DBP for each vitamin D group compared to placebo.

^4^P-value represents overall difference in means across treatment groups.

^ab^SBP was different between vitamin D group compared to placebo; estimated using the contrast of margins (p<0.01)

^cd^DBP was different between vitamin D group compared to placebo; estimated using the contrast of margins (p<0.01)

| **Table S10. Effect of vitamin D supplementation**^1^ **on changes in maternal blood pressure in intervals across pregnancy by limiting to term births (n=1110).** | | | | | | | |
| --- | --- | --- | --- | --- | --- | --- | --- |
|  | **SBP Δ per week (mm Hg)**^2^ | | |  | **DBP Δ per week (mm Hg)**^2^ | | |
| **Gestational Age** | **β** | **95% CI** | **P-Value**^3^ |  | **β** | **95% CI** | **P-Value**^3^ |
| **24 to 30 weeks** |  |  |  |  |  |  |  |
| Placebo | ref |  |  |  | ref |  |  |
| 4200 IU/wk | -0.011 | (-0.367, 0.343) | 0.951 |  | -0.065 | (-0.352, 0.221) | 0.656 |
| 16800 IU/wk | -0.027 | (-0.388, 0.334) | 0.884 |  | -0.059 | (-0.351, 0.233) | 0.693 |
| 28000 IU/wk | 0.040 | (-0.270, 0.351) | 0.798 |  | 0.060 | (-0.192, 0.310) | 0.644 |
| **30 to 36 weeks** |  |  |  |  |  |  |  |
| Placebo | ref |  |  |  | ref |  |  |
| 4200 IU/wk | 0.121 | (-0.212, 0.454) | 0.476 |  | 0.084 | (-0.185, 0.353) | 0.539 |
| 16800 IU/wk | -0.016 | (-0.352, 0.320) | 0.925 |  | 0.057 | (-0.214, 0.328) | 0.680 |
| 28000 IU/wk | 0.181 | (-0.111, 0.471) | 0.222 |  | 0.187 | (-0.047, 0.422) | 0.118 |
| **>36 weeks** |  |  |  |  |  |  |  |
| Placebo | ref |  |  |  | ref |  |  |
| 4200 IU/wk | 0.193 | (-0.483, 0.869) | 0.577 |  | -0.012 | (-0.560, 0.535) | 0.965 |
| 16800 IU/wk | 0.047 | (-0.654, 0.334) | 0.896 |  | 0.188 | (-0.379, 0.755) | 0.516 |
| 28000 IU/wk | -0.240 | (-0.842, 0.362) | 0.434 |  | -0.013 | (-0.500, 0.474) | 0.959 |

CI, confidence interval; DBP, diastolic blood pressure; IU, international unit; SBP, systolic blood pressure.

^1^Vitamin D doses were administered weekly within a randomized controlled trial.

^2^Mixed-effects models with linear spline knots at 30, and 36 weeks gestation were used to estimate effect of vitamin D on changes in SBP or DBP. Models included spline terms for gestational age; treatment groups; and interactions terms between treatment group and gestational age spline terms. Models were fit to test the effect of each vitamin D treatment group on SBP or DBP with reference to placebo. Models were adjusted for baseline SBP or DBP and gestational age at enrollment. Estimates for vitamin D groups are the coefficients for the interaction terms between treatment group and gestational age spline terms which represent the mean change in SBP or DBP between vitamin D groups compared to placebo at specified timepoints. Interclass correlation coefficient: SBP, 0.399; DBP, 0.413.

^3^P-value is for the interaction term between treatment group and gestational age spline terms.

Figure S1. Systolic and diastolic blood pressure across pregnancy from enrollment until delivery among women in the placebo group. The fit line represents LOWESS (Locally Weighted Scatterplot Smoothing) curves.

**References**

1. Roth DE, Morris SK, Zlotkin S, et al. Vitamin D Supplementation in Pregnancy and Lactation and Infant Growth. *N Engl J Med* 2018;379:535-546.
